# DDX5/p68 RNA helicase expression is essential for initiating adipogenesis

**DOI:** 10.1186/s12944-015-0163-6

**Published:** 2015-12-04

**Authors:** Nardev Ramanathan, Nicole Lim, Colin L. Stewart

**Affiliations:** Developmental and Regenerative Biology Laboratory, Institute of Medical Biology, #06-06, 8A Biomedical Grove, Singapore, 138648 Republic of Singapore; School of Biological Sciences, College of Science, Nanyang Technological University, Singapore, 637551 Republic of Singapore; Present address: Davos Life Science Pte Ltd, 3 Biopolis Drive, #04-19, Synapse, Singapore, 138623 Republic of Singapore

**Keywords:** DDX5/p68, Adipogenesis, RNA helicase

## Abstract

**Background:**

DDX5/p68 RNA helicase is a member of the DEAD (Asp-Glu-Ala-Asp) box proteins. Apart from RNA unwinding, DDX5 is an important transcriptional factor and co-activator in cell proliferation and differentiation.

**Findings:**

Here, we have characterised the role of DDX5 in adipogenesis in 3T3-L1 cells using siRNA mediated suppression. Transient inhibition of *Ddx5* mRNA expression at the start of adipogenesis impairs the differentiation programme even when DDX5 expression is restored later in adipogenesis. However transient suppression of *Ddx5* at the later stages of adipogenesis do not impair adipogenesis or triglyceride accumulation suggesting *Ddx5* expression is dispensable in a mature adipocyte.

**Conclusion:**

These results implicate DDX5 as a crucial factor involved in the complex transcriptional cascade of events that regulate adipogenesis and essential to the initiation of adipogenesis.

## Introduction

DEAD (Asp-Glu-Ala-Asp) Box Protein 5 (DDX5), or p68 RNA helicase is a multifunctional nuclear factor, first identified by its immunological cross-reactivity to a monoclonal antibody to the large T antigen of simian virus 40 [1]. DDX5 is a prototypical member of the DEAD/H-box protein family. The DEAD box family is characterized by a region of nine conserved amino acid motifs including Asp-Glu-Ala-Asp (DEAD) which are required in cellular functions such as pre-mRNA processing and ribosome biogenesis [2]. Co-purification of DDX5 with spliceosomes suggested a role in RNA splicing and this was subsequently confirmed when DDX5 was shown to be an essential splicing protein acting at the U1 snRNA-5′ splice site [3]. More recently, DDX5 has been reported to co-activate several transcription factors in proliferation and differentiation that are themselves highly regulated, including the estrogen receptor α (ERα), tumour suppressor p53, androgen receptor (AR), myogenic regulator MyoD, and the osteoblast differentiation factor Runx2 [4]. DDX5 has been hinted at to be involved in adipogenesis in one report [5], although with rudimentary characterisation. Here we sought to clarify the requirement of DDX5 in adipogenesis using a transient knockdown strategy.

## Materials and methods

### Cell culture and adipogenesis

3T3-L1 murine preadipocytes and C3H10T1/2 murine mesenchymal stem cells were cultured in high glucose (4500 mg/L) Dulbecco’s modified Eagle medium (DMEM) supplemented by 10 % heat-inactivated Newborn Calf Serum (NCS) (Sigma-Aldrich N4637) as described [6]. Two days after reaching 100 % confluence, cells were induced to differentiate into adipocytes in high glucose DMEM supplemented with 10 % Fetal Calf Serum (FCS) as described [6]. Oil Red O staining of lipid accumulation was performed as described [6] and triglyceride levels were quantified using a triglyceride quantification kit (Abcam, ab65336) according to the manufacturer’s protocol.

### RNA isolation and quantitative real-time PCR (qRT-PCR)

RNA extraction and purification was done using the RNeasy Mini Kit (Qiagen, #74106) together with DNase digestion using the RNase-free DNase digestion kit (Qiagen, #79254) and was performed according to the manufacturer’s instructions. Purified RNA was reverse transcribed as previously described [7]. Reactions were performed using either Fast Taqman® master mix with Taqman® primers or probes (ABI Biosystems) or SYBR® green master mix (ABI Biosystems) and primers (Table 1). Expression of all the genes were normalized to the housekeeping gene Cyclophilin A *(CycloA).*

**Table 1:** SYBR green primers used for quantitative real-time PCR

Gene	Forward primer	Reverse primer
*CycloA*	TTCCTCCTTTCACAGAATTATTCCA	CCGCCAGTGCCATTATGG
*Pparg*	GATGCACTGCCTATGAGCACT	AGAGGTCCACAGAGCTGATTC
*Cebpb*	AAGCTGAGCGACGAGTACAAGA	GTCAGCTCCAGCACCTTGTG
*Lmna*	GGAGGAGCTTGACTTCCAGAAG	CCACAAGCCGCGTCTCAT

### Western blot analysis

Cell lysis and western blotting was performed as described [7]. Antibodies used for western blotting were against PPARγ (E8)(Santa Cruz, sc-7273, 1:1000), DDX5 (PAb204, kind gift from Frances-Fuller Pace, University of Dundee, 1:500) and GAPDH (FL-335, sc-25778, 1:2000).

### siRNA mediated suppression of gene expression

Cells were treated with 20 μm of either a control non-targeting siRNA or SMARTpool: ON-TARGETplus (GE Lifesciences) siRNA targeted against *Ddx5*, *Pparg* or *Cebpb* (Table 2) using Lipofectamine RNAiMAX (Life Technologies) as described [8].

**Table 2:** The SMARTpool siRNA ON-TARGETplus mouse siRNA (Thermo Scientific) designed to specifically target mouse *Ddx5*, *Pparg* and *Cebpb*. The SMARTpool siRNA consists of four individual duplexes designed to obtain a high level of gene silencing. The non-targeting siRNA is a negative control siRNA with at least four mismatches to any human mouse or rat gene

siRNA	siRNA details	Target sequence
*Ddx5* (13207)	*Ddx5* -J-065333-09	CAGUAAAGUUUUCGGGUUA
	*Ddx5* -J-065333-10	AAAUAAGACCUGAUAGGCA
	*Ddx5* -J-065333-11	GCUGAAUAUUGUCGAGCUU
	*Ddx5* -J-065333-12	ACAUAAAGCAAGUGAGCGA
*Pparg* (19016)	*Pparg* -J-040712-05	CGAAGAACCAUCCGAUUGA
	*Pparg* -J-040712-06	ACCCAAUGGUUGCUGAUUA
	*Pparg* -J-040712-07	UCACAAUGCCAUCAGGUUU
	*Pparg* -J-040712-08	CGACAUGAAUUCCUUAAUG
*Cebpb* (12608)	*Cebpb* -J-043110-09	GAGCGACGAGUACAAGAUG
	*Cebpb* -J-043110-10	CCUUUAGACCCAUGGAAGU
	*Cebpb* -J-043110-11	GCACCCUGCGGAACUUGUU
	*Cebpb* -J-043110-12	GAAAAGAGGCGUAUGUAUA
Non-targeting siRNA #2	D-001810-02-05	UGGUUUACAUGUUGUGUGA

### Cell viability assay

Cells were treated with WST-1 reagent using the WST-1 Cell Viability Assay Kit (Cayman Chemicals, #10008883) according to the manufacturer’s protocol. Briefly, preadipocytes were seeded at a density of 10^4^ cells/cm^2^ in a 96-well plate in triplicates. Cells were normalized to untreated cells. Bleomycin (Sigma-Aldrich, B8416) was used as a negative control (cytotoxic agent) at 200 μg/ml. Both siRNA treatment and bleomycin treatment were administered for 48 h.

## Results

**Fig. 1:**
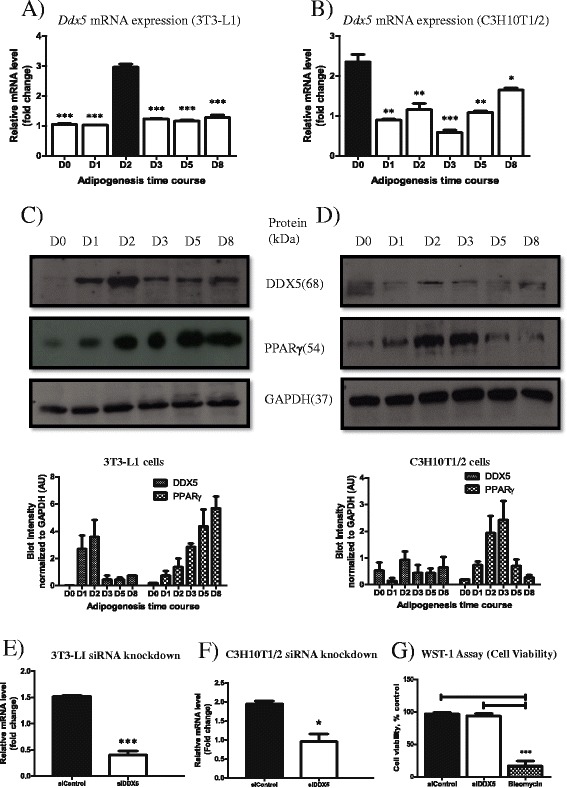
mRNA and protein expression of DDX5 assayed in 3T3-L1 preadipocytes and C3H10T1/2 mesenchymal stem cells induced to undergo adipogenesis for 8 days in vitro. mRNA expression of *Ddx5* in (**a**) 3T3-L1 and (**b**) C3H10T1/2 cells differentiated for 8 days in vitro. Black bars indicate the peak of mRNA expression. Protein expression by western blotting for DDX5, PPARγ and GAPDH in (**c**) 3T3-L1 and (**d**) C3H10T1/2 cells differentiated for 8 days in vitro. Western blot bands were quantified using ImageJ. Transient knockdown using siRNA were performed on (**e**) 3T3-L1 and (**f**) C3H10T1/2 cells. **g** Cell viability assay using WST-1 was performed using cells treated with control siRNA (siControl), siRNA against *Ddx5* (siDDX5) and bleomycin and compared to untreated cells. Data are represented as % cell viability compared to untreated cells. *p < 0.05, **p < 0.01, ***p < 0.001. Experiments were done in triplicates and error bars indicate SEM (*n* = 3). mRNA expression data and western blot densitometry were normalized to *CycloA* and GAPDH expression respectively

*DDX5 expression in differentiating adipocytes*- We investigated the expression of DDX5 during adipogenesis in two widely used preadipocytic lines, 3T3-L1 and C3H10T1/2 mesenchymal stem cells. We differentiated these lines for 8 days in vitro and assessed the mRNA and protein expression of DDX5. We observed that the mRNA expression of *Ddx5* in 3T3-LI cells peaked at day 2 of differentiation (Fig. 1a) whereas *Ddx5* was most strongly expressed before adipogenic induction (D0) in C3H10T1/2 cells (Fig. 1b). However, at the protein level, DDX5 peaked at D2 of differentiation in both these cell lines (Fig. 1c, d). The expression of peroxisome-proliferator activated receptor γ (PPARγ), a critical regulator of adipogenesis, that is induced during differentiation, confirmed functional adipogenesis (Fig. 1c, d).

**Fig. 2:**
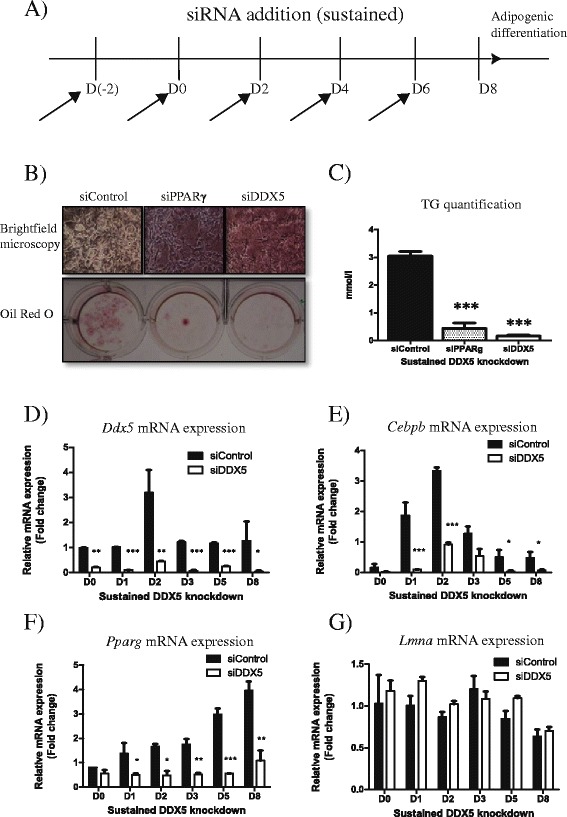
Sustained knockdown of *Ddx5* in 3T3-L1 cells differentiated for 8 days in vitro. (**a**) Cells were treated with siRNA continuously up to day 6 of differentiation and (**b**) viewed under a microscope and also stained for lipid accumulation using Oil Red O Stain. (**c**) Triglyceride accumulation was quantified and mRNA expression of (**d**) *Ddx5*, (**e**) *Cebpb*, (**f**) *Pparg* and (**g**) *Lmna* were investigated. All experiments were done in triplicates and statistical significance are denoted as follows: *p < 0.05, **p < 0.01, ***p < 0.001. Error bars indicate SEM (*n* = 3). mRNA expression data were normalized to *CycloA* expression respectively

*DDX5 is required for adipogenesis* –Next, we investigated the consequences of supressing *Ddx5* expression using siRNA. We obtained an >80 % reduction in transcript levels using siRNA targeted against *Ddx5* in 3T3-L1 cells but only a ~55 % reduction in C3H10T1/2 cells compared to cells treated with control siRNA (Fig. 1e, f). Cell viability was assessed to investigate if the Ddx5 knockdown impaired viability. We observed that Ddx5 knockdown did not significantly impair viability (>90 % viability) when compared to untreated cells (Fig. 1g). Given the greater knockdown observed in 3T3-L1 cells, subsequent knockdown experiments were performed on 3T3-L1 cells to understand how the loss of *Ddx5* affects adipogenesis. *Ddx5* was suppressed throughout adipogenesis in a sustained and systematic manner by the addition of siRNA every 2 days starting from 2 days before the induction of adipogenesis to day 6 of differentiation (Fig.2a, d). We found that treatment with siRNA against *Ddx5* resulted in impaired triglyceride accumulation compared to treatment with control siRNA, as observed by brightfield microscopy, Oil Red O staining and triglyceride quantification (Fig. 2b, c). Cells were also treated with siRNA against *Pparg* as a positive control, as it is well established that disruption of *Pparg* expression blocks triglyceride accumulation and adipogenesis [9]. Since *Ddx5* suppression impairs triglyceride accumulation, we determined if this was due to impaired adipogenesis. We assessed mRNA expression of *Ddx5*, CCAAT enhancer binding protein β (*Cebpb*), a transcription factor critical at the early stages of adipogenesis [9], *Pparg*, a critical regulator that maintains the mature adipocytic state [9], and *Lmna* whose expression is relatively stable during adipogenesis and in fully differentiated cells [10]. *Ddx5* expression was significantly suppressed by siRNA treatment throughout adipogenesis as expected (Fig. 2d). Both *Cebpb* and *Pparg* expression were also significantly reduced (Fig. 2e, f), suggesting that both these early and later regulators of adipogenesis are reduced by the knockdown of *Ddx5. Lmna* expression was unaffected by the *Ddx5* inhibition (Fig. 2g) suggesting that the *Ddx5* suppression particularly affects genes that regulate adipogenesis.

**Fig. 3:**
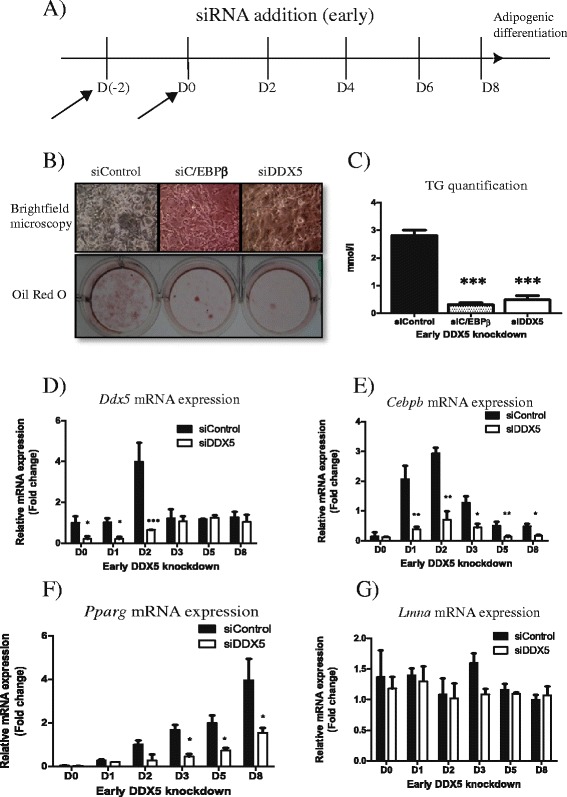
Early knockdown of *Ddx5* in 3T3-L1 cells differentiated for 8 days in vitro. (**a**) Cells were treated with siRNA only at D(−2) and D0 of differentiation and (**b**) viewed microscopically and also stained for lipid accumulation using Oil Red O stain. (**c**) Triglyceride accumulation was quantified and mRNA expression of (**d**) *Ddx5*, (**e**) *Cebpb*, (**f**) *Pparg* and (**g**) *Lmna* were investigated. All experiments were performed in triplicates and statistical significance are denoted as follows: *p < 0.05, **p < 0.01, ***p < 0.001. Error bars indicate SEM (*n* = 3). mRNA expression data were normalized to *CycloA* expression respectively

*DDX5 is crucial at the early stages of adipogenesis* – Given the peak of expression at D2 of differentiation (Fig. 1a, c) and since *Ddx5* is required for adipogenesis (Fig. 1e, f), we investigated the possibility of *Ddx5* being crucial to the early stages of adipogenesis. We investigated this by transiently suppressing *Ddx5* mRNA expression at the initial stages of adipogenesis: 2 days before adipogenic induction of adipogenesis (D-2) and at the same time as adipogenic induction (D0) (Fig. 3a, b). Differentiation was maintained for 8 days, however triglyceride accumulation was severely impaired, indicating defective adipogenesis (Fig. 3b, c). *Cebpb* is a well-established crucial factor in the early stages of adipogenesis [9], and was analyzed to further understand how the loss of *Ddx5* affects early adipogenesis. While *Ddx5* was only transiently suppressed at the early stages of differentiation (Fig. 3d), the expression of both *Cebpb* and more interestingly *Pparg,* a gene expressed much later in adipogenesis, were impaired compared to cells treated with control siRNA (Fig. 3e, f), whereas the expression of *Lmna* was unaffected (Fig. 3g). *Cebpb* induces the expression of *Pparg* [9] and this may explain, at least in part, the suppression of *Pparg* expression.

**Fig. 4:**
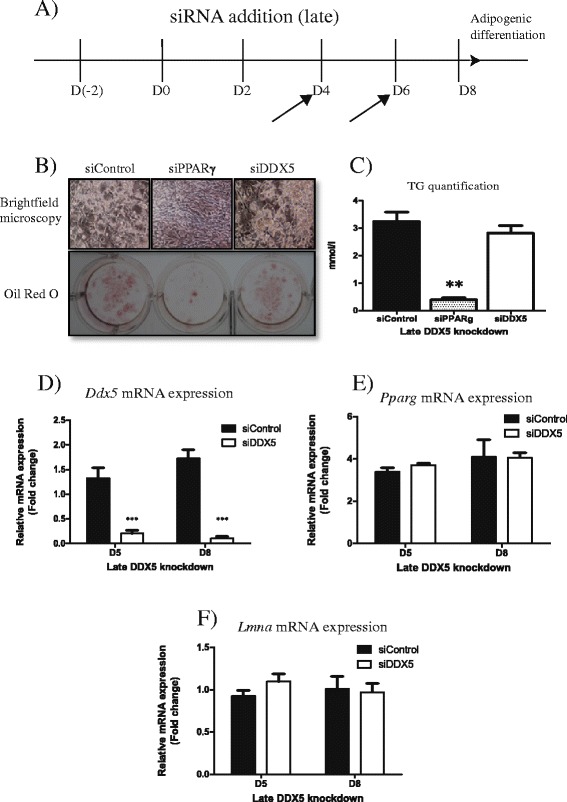
Late knockdown of *Ddx5* in 3T3-L1 cells differentiated for 8 days in vitro. (**a**) Cells were treated with siRNA only at D4 and D6 of differentiation and (**b**) viewed microscopically and also stained for lipid accumulation using Oil Red O stain. (**c**) Triglyceride accumulation was quantified and mRNA expression of (**d**) *Ddx5*, (**e**) *Pparg* and (**f**) *Lmna* were described. All experiments were done in triplicates and statistical significance are denoted as follows: *p < 0.05, **p < 0.01, ***p < 0.001. Error bars indicate SEM (*n* = 3). mRNA expression data were normalized to *CycloA* expression respectively

*DDX5 is not required in mature adipocytes* – Since *Ddx5* was required during the early stages of adipogenesis we investigated the requirement for DDX5 at the later stages of adipogenesis. Differentiating 3T3-LI adipocytes were treated with siRNA at days 4 and 6 of differentiation (Fig. 4a). Prior to this, the cells are differentiated but untreated and so indistinguishable experimentally. Thus data only from D4 onwards would be relevant and meaningful and only these are presented. In addition, analysis of *Cebpb* is also omitted given that its expression is only relevant early in adipogenesis. Cells treated with siRNA targeting *Ddx5* in this manner accumulated triglycerides as well as cells treated with control siRNA (Fig. 4b, c). However treatment with siRNA against *Pparg* impaired the maintenance of the mature adipocyte state, consistent with previous reports [11]. *Ddx5* siRNA suppressed *Ddx5* expression (Fig. 4d) but *Pparg* and *Lmna* levels were unaffected (Fig. 4e, f). *Ddx5* therefore appears to be essential for the initial stages of adipogenesis, but not at the later stages of differentiation and maintenance of the mature adipocyte state.

## Discussion

Here we demonstrate that DDX5/p68 RNA helicase peaks in expression on day 2 of adipogenesis in two different murine cell lines (Fig. 1a–d). These observations led us to speculate that this transient expression is required at the early stage of adipogenesis. The transcriptional pathways controlling adipocyte differentiation are relatively well characterized [12]. Adipogenesis is regulated by a cascade in the expression of a variety of transcription factors where an early wave of factors is induced immediately at the start of differentiation. These factors then activate a second set of transcription factors that are responsible in maintaining the mature and functional adipocyte. Utilising a transient knockdown strategy, we observed that DDX5 is essential during the initial stages of adipogenesis (Fig. 3) but not at later stages (Fig. 4). This suggests a role for DDX5, potentially as a transcriptional co-activator, at the early stages of adipocytic differentiation, placing it in the same category as other well established transcriptional factors such as C/EBPβ/δ and the Kruppel-like factors (KLFs) [9]. In future studies it will be of interest to examine how the loss of Ddx5 affects lipid droplet biology given the increasing evidence of genes expressing lipid droplet associated proteins being implicated in metabolic diseases such as lipodystrophy [13]. This unappreciated role of DDX5 in adipogenesis provides further insight into adipogenic molecular pathways, providing an additional potential target to address metabolic diseases including obesity and type 2 diabetes that are of increasing importance to global public health.
